# Ultrasound-Guided Percutaneous Cryoneurolysis for Perioperative Analgesia Following Major Lower Extremity Amputation: A Randomized, Participant- and Observer-Masked, Sham-Controlled Pilot Study

**DOI:** 10.7759/cureus.53563

**Published:** 2024-02-04

**Authors:** John J Finneran, Alexandra K Schwartz, Paul J Girard, William T Kent, Omar Al-Nouri, Andrea Trescot, Brian M Ilfeld

**Affiliations:** 1 Anesthesiology, University of California San Diego, San Diego, USA; 2 Orthopedic Surgery, University of California San Diego, San Diego, USA; 3 Vascular Surgery, University of California San Diego, San Diego, USA; 4 Pain Medicine, Florida Pain Relief Group, Tampa, USA

**Keywords:** femoral nerve, sciatic nerve, phantom limb, regional anesthesia, cryoneurolysis, nerve block, amputation

## Abstract

Background: Extremity amputations are associated with pain in both the residual limb and the phantom limb. This pain, which is often debilitating, may be prevented by excellent perioperative pain control. Ultrasound-guided percutaneous cryoneurolysis is an analgesic modality offering pain control for weeks or months following surgery. This treatment has not been compared to the sham procedure for large nerves (e.g., femoral and sciatic) to provide preoperative analgesia. We therefore conducted a randomized, controlled pilot study to evaluate the use of this modality for the treatment of pain following amputation to (1) determine the feasibility of and optimize the study protocol for a subsequent definitive clinical trial; and (2) estimate analgesia and opioid reduction within the first postoperative weeks.

Methods: A convenience sample of seven patients undergoing lower extremity amputation were randomized to receive either active cryoneurolysis or a sham procedure targeting the sciatic and femoral nerves in a participant-masked fashion. This was a pilot study with a relatively small number of participants, and therefore the resulting data were not analyzed statistically.

Results: Compared to the participants who received sham treatment (n=3), those who underwent active cryoneurolysis (n=4) reported lower pain scores and decreased opioid consumption at nearly all time points between days one and 21 following amputation.

Conclusions: Ultrasound-guided percutaneous cryoneurolysis of the femoral and sciatic nerves prior to lower extremity amputation appears feasible and potentially effective. The data from this pilot study may be used to power a definitive randomized clinical trial.

## Introduction

Over one million Americans are currently living with an amputated limb. This number has been steadily increasing and is likely to exceed three million by 2050 [1]. Phantom pain, which is pain experienced in the area of the limb that is no longer present, is associated with significant disability, is multifactorial in etiology, and is difficult to treat once established [2]. Perioperative epidural local anesthetic and/or intravenous opioid infusion is associated with decreased intensity, prevalence, and frequency of chronic pain following limb amputation [3]. However, these analgesic modalities require hospitalization and are associated with various complications, such as hypotension, urinary retention, and/or respiratory depression. Thus, there is a substantial need for an analgesic modality that can provide excellent postoperative analgesia for an extended duration following amputation.

Cryoneurolysis, the use of extreme cold to reversibly ablate individual peripheral nerves, offers the possibility of prolonged analgesia measured in weeks or months with a single perioperative treatment [4]. Exposure to extreme cold (approximately -60ºC) causes peripheral nerve axons to undergo Wallerian degeneration distal to the site of cryoneurolysis application, although the endoneurium, perineurium, and epineurium remain intact [5, 6]. These axons subsequently regenerate at a rate of approximately 1-2 mm/day [7]. Thus, this technique produces a reversible nerve block with a duration measured in weeks or months [4]. However, if a therapeutic temperature resulting in Wallerian degeneration is not reached, the result can be a short-lived neuropraxia and an unpredictable clinical effect [5].

Utilizing ultrasound guidance for probe placement, cryoneurolysis can target deep nerves percutaneously with a technique similar to conventional ultrasound-guided peripheral nerve blocks [4]. Applied to very small nerves (e.g., intercostal, lateral femoral cutaneous, infrapatellar branch of the saphenous) for postoperative analgesia, this modality not only decreases acute pain following surgery but also the incidence of pain up to one year following surgery [8-10]. However, while small nerves appear to be easily treated with percutaneous cryoneurolysis, the relatively small zone of adequate treatment (approximately -60ºC required for Wallerian degeneration) formed with most percutaneous probes (1 mm or less) usually will not fully encompass larger nerves [5, 6, 11]. This failure to adequately encompass the cross-sectional area of larger nerves may at least partially explain the negative results of a recent study examining the use of ultrasound-guided percutaneous femoral and sciatic nerve cryoneurolysis for the treatment of established phantom pain [12].

Given the small zone of adequate treatment associated with most cryoneurolysis probes and the comparatively large cross-sectional area of peripheral nerves such as the femoral and sciatic nerves, multiple freeze treatments may be required at different locations around the nerve to adequately treat larger nerves and induce Wallerian degeneration. We therefore undertook this randomized, sham-controlled pilot study to assess the feasibility of perioperative sciatic and femoral cryoneurolysis, moving the probe tip between freeze cycles, for pain control following lower extremity amputations. The goals of the present study were to optimize the study protocol and collect data to power a subsequent, definitive clinical trial.

## Materials and methods

Enrollment

This single-center, randomized, sham-controlled, parallel-group pilot study adhered to Good Clinical Practice quality standards and ethical guidelines defined by the Declaration of Helsinki. Prospective study protocol approval as well as ongoing data and safety oversight were conducted by the University of California San Diego Institutional Review Board (IRB approval number: #180863), San Diego, CA. The study was prospectively registered at clinicaltrials.gov (NCT03578237; Principal Investigator Brian M. Ilfeld, MD, MS; date of registration: July 6, 2018) prior to any participant enrollment. Written, informed consent was obtained from all participants prior to enrollment or any study interventions. Beginning January 3, 2019, and ending January 28, 2022, participation was offered to adults (18 years of age or older) scheduled to undergo a lower extremity amputation for any indication with planned femoral and sciatic nerve blocks for postoperative analgesia who agreed to be available by telephone for 11 follow-up phone calls over the subsequent year. Exclusion criteria included chronic opioid use, morbid obesity, infection at the cryoneurolysis site, incarceration, pregnancy, and individuals with a medical condition that is a contraindication to cryoneurolysis (e.g., cryoglobulinemia, cold urticaria, Reynaud’s syndrome) [4].

Preoperative procedures

After applying standard American Society of Anesthesiologists monitors, participants were positioned lateral or prone, and oxygen was administered via facemask. If required for anxiolysis, intravenous fentanyl and midazolam were titrated for patient comfort. However, careful attention was paid to avoid over-sedation and maintain spontaneous ventilation. The sciatic nerve was identified in the short axis using a 13- to 6-MHz linear ultrasound transducer (HFL38, Edge-II, SonoSite, Bothell, WA) 3-5 centimeters proximal to the popliteal fossa. As previously described, a 17-gauge (G) Tuohy needle was inserted adjacent to the sciatic nerve, and a 19-gauge flexible, single-orifice perineural catheter was inserted under ultrasound guidance. Lidocaine, 2.0%, with 5-10 µg/mL epinephrine (20 mL) was injected in divided doses through the catheter, which was then secured with clear, occlusive dressings and taped up the lateral thigh [13]. After securing the catheter, subjects returned to the supine position, and the femoral nerve was identified at the level of the inguinal crease with a liner ultrasound transducer. After prepping the needle entry site with chlorhexidine and using an in-plane, ultrasound-guided technique, a 20-G Tuohy needle was advanced adjacent to the femoral nerve. Ropivacaine 0.5% (15-20 mL) was injected to provide circumferential coverage of the nerve. Approximately 20 minutes following the nerve block procedure, loss of temperature discrimination to ice in the femoral and sciatic distributions was confirmed.

Study intervention

**Figure 1:**
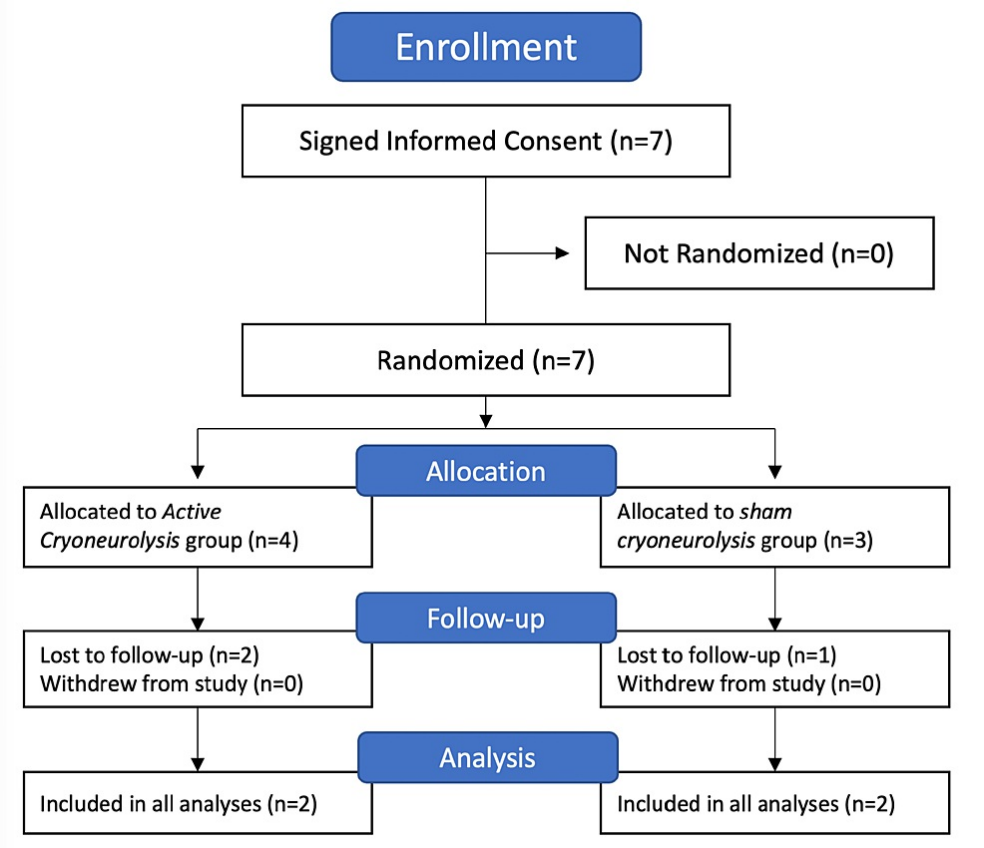
Consolidated Standards of Reporting Trials (CONSORT) diagram of the study procedure

Either in the preoperative area after confirming successful local anesthetic block of the femoral and sciatic nerves or in the recovery area (if there was any chance an intraoperative decision would be made to not proceed with amputation), participants were randomly allocated to one of two treatments: (1) active cryoneurolysis or (2) sham (Figure 1).

**Figure 2:**
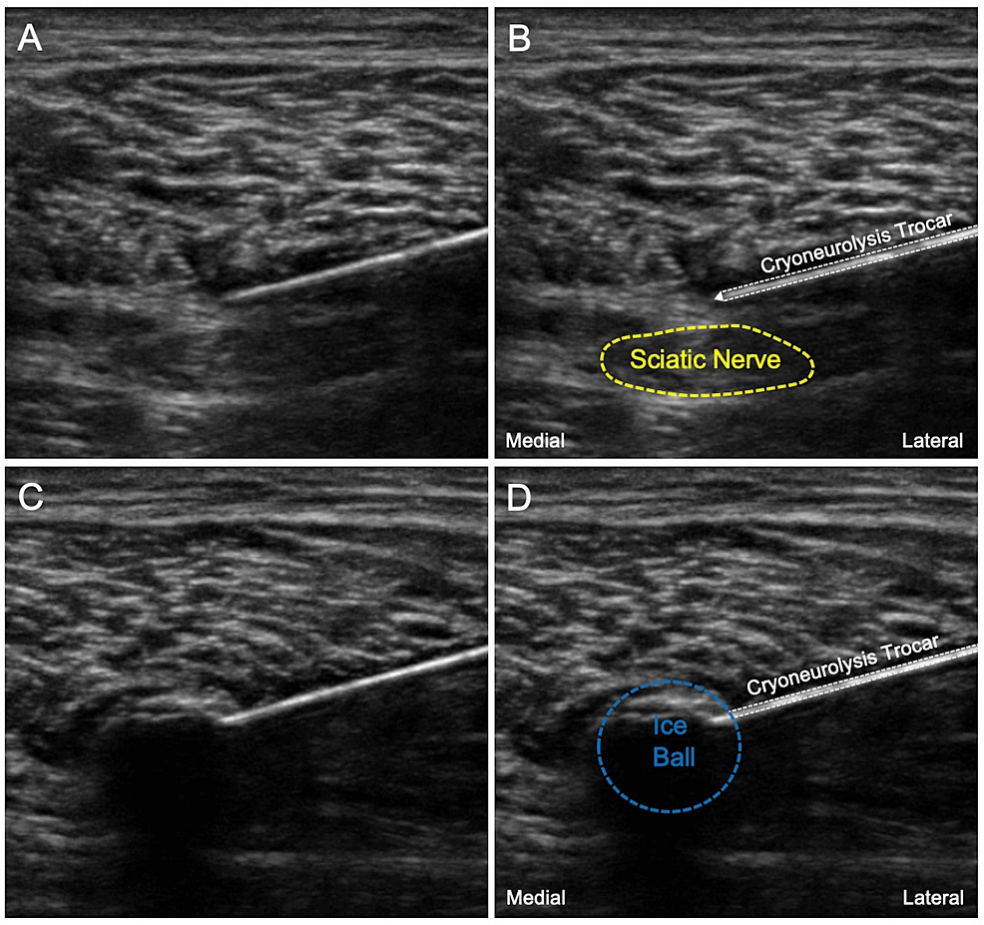
(A) The sciatic nerve is identified proximal to its bifurcation, and the cryoneurolysis probe is inserted adjacent to the nerve. (B) The sciatic nerve (yellow) and cryoneurolysis trocar (white) are labeled. (C) The frozen tissue (ice ball) is visualized enveloping the sciatic nerve. (D) Cryoneurolysis trocar (white) and ice ball (blue) are labeled.

Computer-generated randomization lists (blocks of four) were used by the university’s Investigational Drug Service to create sealed, opaque randomization envelopes enclosing the treatment group assignment (Statmate; GraphPad Software, La Jolla, CA). The investigator administering the study intervention opened the randomization envelope. Therefore, investigators, subjects, and clinical staff were masked to treatment group assignment, with the only exception being the unmasked individual who performed the procedure (and did not have subsequent contact with the participant). Participants were again positioned either lateral or prone, and the sciatic nerve was visualized with the same linear ultrasound transducer, 2-3 cm proximal or distal to the perineural catheter location (to avoid damaging the perineural catheter while advancing the cryoneurolysis probe). After anesthetizing the skin with lidocaine 1% (1-2 mL) and prepping with chlorhexidine gluconate, a 14G angiocatheter was inserted as an introducer, and then a 16G cryoneurolysis probe, connected to a console cryoneurolysis machine (PainBlocker, Epimed International, Dallas, TX), was advanced to a point adjacent to the sciatic nerve (Figures 2A-2B).

The cryoneurolysis machine was activated for three cycles, each consisting of two minutes on (freeze) and one minute off (to allow for thawing in the active treatment group). The probe was repositioned between the cycles to cover the entire cross-section of the nerve (for the active treatment group).

Active Group

The ice ball at the tip of the cryoneurolysis probe was viewed as enveloping at least 1 cubic centimeter of the sciatic nerve (Figures 2C-2D).

Sham Group

The sham probe vented the nitrous oxide at its proximal end, and therefore no freezing occurred at the distal tip. The difference between the active and sham probes is imperceptible to the patient, although the physician operator is aware of the treatment assignment due to the lack of ice ball visualization by ultrasound in the latter. Therefore, study participants remained masked to treatment group assignments.

The cryoneurolysis procedure was repeated for the femoral nerve at a location approximately 5 cm distal to the inguinal crease to limit the extended duration of quadriceps weakness.

Intraoperative procedures

Participants received either general endotracheal anesthesia consisting of sevoflurane in a nitrous oxide-air mixture or propofol sedation at the discretion of the intraoperative anesthesia team. Intraoperative opioids were administered by the same team, as needed.

Postoperative procedures

Following surgery, participants were transferred to the recovery area and subsequently discharged to either the orthopedic ward or home at the discretion of the surgical team. A perineural infusion using an electronic pump of ropivacaine 0.2% administered via the sciatic catheter (basal 6 mL/hour; 4 mL demand bolus; 30-minute lockout) was initiated in the recovery area. For patients discharged home, a three-day supply of ropivacaine was provided with a disposable pump. For participants admitted following surgery, the infusion was continued for the duration of the hospitalization or as determined by the hospital’s acute pain service. Supplementing the ropivacaine infusion and study intervention, participants received an analgesic regimen consisting of scheduled oral acetaminophen with oral oxycodone (5-15 mg), as needed. If admitted postoperatively, ultimate discharge readiness was determined by the surgical team. 

Outcomes measures

All study-related follow-up was done by an investigator blinded to treatment group allocation. The first two items of the Brief Pain Inventory-worst and average pain at the amputation donor site using a Numeric Rating Scale (NRS) of 0-10, with 0 corresponding to “no pain” and 10 corresponding to “worst imaginable pain"-were collected on postoperative days (POD) one, two, three, four, seven, 14, and 21, as well as at months one, three, six, and 12. Additionally, at months one, three, six, and 12, participants completed the Brief Pain Inventory-Short Form and were asked to differentiate average, worst, least, and current pain scores for both the wound (residual limb) pain and phantom pain. Opioid consumption was recorded in the electronic medical record while the participants remained hospitalized. For the time points following, all participant follow-up was achieved by telephone.

Statistical analysis

The objectives of this pilot study were to investigate the feasibility of cryoneurolysis for pain control following limb amputation, optimize the protocol, and collect data to power a subsequent, definitive clinical trial. As such, a convenience sample was used, and statistics were not applied to the results given the small sample size and lack of an a priori statistical plan.

## Results

Seven subjects were enrolled between January 3, 2019, and January 28, 2022 (Table 1), had successful local anesthetic blocks, underwent below-knee amputation, and received the study intervention (four subjects received active cryoneurolysis and three subjects received sham).

**Table 1:** Demographics of the study group Values are reported as mean ± standard deviation or absolute number (percentage of treatment group) as appropriate.

Factor	Active cryoneurolysis (N=4)	Sham (N=3)
Age (years)	56 ± 15	57 ±10
Male – number (%)	4 (100)	3 (100)
BMI (kg/m^2^)	26.1 ± 6.0	26.1 ± 1.2

**Figure 3:**
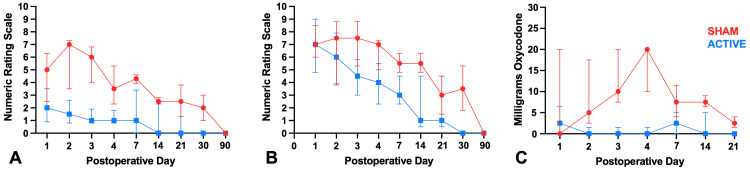
(A) Average and (B) worst pain and (C) opioid consumption (median, 25th percentile, 75th percentile) in participants who received active cryoneurolysis (blue) vs. sham procedure (red) during the first 90 days following amputation.

At nearly all time points within the first three weeks, participants randomized to active cryoneurolysis experienced lower average and maximum pain scores compared to those who underwent the sham procedure (Figure 3).

The participants in the active group also required fewer opioid analgesics on nearly all days during this period compared to the sham group (Figure 3). Follow-up was challenging in this patient population after the first three weeks (Table 2).

**Table 2:** Results of the study Average and worst phantom pain and wound pain at months one, three, six, and 12 following amputation. As follow-up for this patient population proved challenging, the number of participants (n) successfully contacted at each time point is provided. NRS: Numeric Rating Scale

Outcome	Active cryoneurolysis (n=4)	Sham (n=3)
Average phantom pain (NRS 0-10)		
Month 1	0 ± 0 (n = 2)	1 ± 1 (n = 2)
Month 3	0 (n= 1)	1 ± 1 (n = 2)
Month 6	0 ± 0 (n = 2)	-
Month 12	0 ± 0 (n = 2)	-
Worst phantom pain (NRS 0-10)		
Month 1	0 ± 0 (n = 2)	3 ± 5 (n = 2)
Month 3	0 (n = 1)	6 ± 1 (n = 2)
Month 6	0 ± 0 (n = 2)	-
Month 12	0 ± 0 (n = 2)	-
Average wound pain (NRS 0-10)		
Month 1	0 ± 0 (n = 2)	2 ± 3 (n = 2)
Month 3	0 (n = 1)	0 ± 0 (n = 2)
Month 6	0 ± 0 (n = 2)	-
Month 12	0 ± 0 (n = 2)	-
Worst wound pain (NRS 0-10)		
Month 1	0 ± 0 (n = 2)	4 ± 5 (n = 2)
Month 3	0 (n = 1)	0 ± 0 (n = 2)
Month 6	0 ± 0 (n = 2)	-
Month 12	0 ± 0 (n = 2)	-

Although few participants could be reached following the first postoperative month, neither of the two active participants who could be reached at months one, six, and 12 reported any surgical wound pain, phantom limb pain, or opioid consumption (Table 2). The datasets generated during this study are available from the corresponding author upon reasonable request.

Protocol deviations

One subject in the active cryoneurolysis group opted to receive only single-injection nerve blocks rather than a continuous sciatic block after signing his informed consent. Thus, the lidocaine for his sciatic nerve block was replaced with ropivacaine 0.5% with 2.5 mcg/mL epinephrine (20 mL).

## Discussion

This randomized, double-masked, sham-controlled pilot study suggests that ultrasound-guided percutaneous cryoneurolysis of the femoral and sciatic nerves improves analgesia and decreases opioid requirements following below-knee lower extremity amputation. It demonstrates the feasibility of treating relatively large nerves with perioperative percutaneous cryoneurolysis, tests a protocol for a future definitive trial, and provides data to help power such an investigation. Although these participants were difficult to contact after the one-month time point, no subject who received femoral and sciatic cryoneurolysis reported experiencing phantom/residual limb pain or the need for opioids following the one-month acute pain phase of recovery.

Cryoneurolysis offers benefits compared to both opioids and local anesthetics for postoperative analgesia. The most significant benefit is the duration of action, which is measured in weeks to months with a single application [4]. Opioid analgesics are associated with systemic side effects that include nausea, vomiting, sedation, and respiratory depression and have significant potential for tolerance, misuse, dependence, overdose, and diversion [14]. In contrast, cryoneurolysis is generally free of these systemic side effects [4]. Continuous peripheral nerve blocks offer the potential for extended-term postoperative analgesia without the systemic side effects associated with opioids. However, the duration of ambulatory continuous peripheral nerve blocks is generally limited to several days due to the size of the local anesthetic reservoir and the potential for catheter site infection, which increases in conjunction with the duration of therapy [15-17]. Continuous peripheral nerve blocks also carry the potential for local anesthetic systemic toxicity and catheter dislodgment. Neither of these complications is possible with cryoneurolysis, as no drug is injected nor is there a catheter left in situ [4, 15-17].

In the present study, the timing of the cryoneurolysis procedure relative to surgery (preoperative vs. postoperative) was based on the likelihood of an intraoperative decision by the surgical team not to proceed with amputation. Cryoneurolysis may produce both sensory and motor blocks with a duration of several months [4]. Thus, it is imperative that this procedure only be performed preoperatively if there is no chance of the surgeon choosing a plan different from amputation in the operating room. Additionally, the site of cryoneurolysis must be chosen based on the level of amputation. For below-knee amputations, patients will be dependent on quadriceps function to participate in rehabilitation. It is critical to perform the cryoneurolysis procedure at least several centimeters distal to the inguinal crease to minimize the effect on quadriceps function. Indeed, in a prior study of cryoneurolysis for treating established phantom pain, a participant with a below-knee amputation was left with quadriceps weakness due to femoral cryoneurolysis at the level of the inguinal crease, resulting in a fall and a traumatic clavicle fracture [12]. 

Limitations

This was a single-center pilot study with a relatively unsuccessful follow-up after three postoperative weeks. No data were available for an a priori sample size estimation, and the convenience sample of seven participants did not permit statistical comparisons between the two treatments. Therefore, the findings should be considered preliminary, requiring confirmation with a subsequent adequately powered prospective clinical trial.

## Conclusions

Ultrasound-guided percutaneous cryoneurolysis of the femoral and sciatic nerves supplementing conventional perioperative analgesics is feasible and appears to improve analgesia for at least one month following below-knee amputation. Cryoneurolysis may be a viable postoperative analgesic option following major limb amputation to treat both acute pain and possibly decrease the incidence and intensity of persistent postoperative phantom and/or residual limb pain. Further investigation in the form of larger randomized controlled trials appears warranted, and these data may serve to help power such investigations.
